# Genomic Characterization of WRKY Transcription Factors Related to Andrographolide Biosynthesis in *Andrographis paniculata*

**DOI:** 10.3389/fgene.2020.601689

**Published:** 2021-01-18

**Authors:** Rongrong Zhang, Zhenzhen Chen, Libing Zhang, Wei Yao, Zhichao Xu, Baosheng Liao, Yaolei Mi, Han Gao, Chunhong Jiang, Lixin Duan, Aijia Ji

**Affiliations:** ^1^Joint Laboratory for Translational Cancer Research of Chinese Medicine of the Ministry of Education of the People's Republic of China, International Institute for Translational Chinese Medicine, School of Pharmaceutical Sciences, Guangzhou University of Chinese Medicine, Guangzhou, China; ^2^Key Laboratory of Bioactive Substances and Resources, Utilization of Chinese Herbal Medicine, Ministry of Education, Institute of Medicinal Plant Development, Chinese Academy of Medical Sciences & Peking Union Medical College, Beijing, China; ^3^State Key Laboratory of Innovative Natural Medicine and TCM Injections, Jiangxi Qingfeng Pharmaceutical Co. Ltd., Ganzhou, China; ^4^School of Chemistry, Chemical Engineering and Life Sciences, Wuhan University of Technology, Wuhan, China

**Keywords:** WRKY, *Andrographis paniculata*, andrographolide biosynthesis, genome-wide, expression patterns

## Abstract

Andrographolide, which is enriched in the leaves of *Andrographis paniculata*, has been known as “natural antibiotic” due to its pharmacological activities such as anti-inflammatory, antimicrobial and antioxidant effects. Several key enzymes in andrographolide biosynthetic pathway have been studied since the genome sequences were released, but its regulatory mechanism remains unknown. WRKY transcription factors proteins have been reported to regulate plant secondary metabolism, development as well as biotic and abiotic stresses. Here, WRKY transcription factors related to andrographolide biosynthesis were systematically identified, including sequences alignment, phylogenetic analysis, chromosomal distribution, gene structure, conserved motifs, synteny, alternative splicing event and Gene ontology (GO) annotation. A total of 58 WRKYs were identified in Chuanxinlian genome and phylogenetically classified into three groups. Moreover, nine WRKY genes underwent alternative splicing events. Furthermore, the combination of binding site prediction, gene-specific expression patterns, and phylogenetic analysis suggested that 7 WRKYs (*ApWRKY01, ApWRKY08, ApWRKY12, ApWRKY14, ApWRKY19, ApWRKY20*, and *ApWRKY50*) might regulate andrographolide biosynthesis. This study laid a foundation for understanding the regulatory mechanism of andrographolide biosynthesis and the improvement and breeding of *Andrographis paniculata* varieties.

## Introduction

*Andrographis paniculata* (Burm.f.) Nees, a well-known traditional herb from the family Acanthaceae, has been used in eastern Asia for thousands of years (Lim et al., 2012). It has a broad spectrum of biological activities, such as anti-inflammatory, antimicrobial, antioxidant, hepatoprotective and hypoglycemic effects (Mishra et al., 2009; Yu et al., 2014). Previous studies have showed that the main active compounds of *A. paniculata* were ent-labdane-related diterpenes (ent-LRDs), including andrographolide (AD), neoandrographolide (NAD) and 14-deoxy-11,12-didehydroandrographolide (DDAD). Their contents varied significantly among different organs, with a marked increase in leaves and lowest in roots (Garg et al., 2015). Among the ent-LRDs, the most abundant constituent is the extremely bitter compound andrographolide, which has been used to treat upper respiratory tract infection, fever and diarrhea in clinical practice in many countries (Lim et al., 2012; Raina et al., 2013). Due to its marked anti-inflammatory and antimicrobial pharmacological activities, andrographolide has been recognized as a “natural antibiotic.” With the increasing clinical demand for andrographolide (Shao et al., 2015), how to efficiently obtain andrographolide by investigating the molecular mechanisms controlling its biosynthesis has received increasing attention. Recently, the genome of *A*. *paniculata* has been completely assembled, and some key genes for the biosynthesis of andrographolide have been discovered (Sun et al., 2019). However, there are no studies about the regulation of andrographolide content by transcription factors, which regulate secondary metabolism at the transcriptional level.

The WRKY TF family possesses ~60 amino acid binding domains and covers three classes (class I to class III) according to the WRKY domain number and the variety of zinc finger motifs (Hussain et al., 2019; Yan et al., 2019). It has been reported to have a significant role in plant secondary metabolism. Several WRKY TFs have been functionally identified as core regulators that regulate the biosynthesis of terpenoids. For example, AaWRKY1 positively regulates the biosynthesis of artemisinin by promoting the expression of the ADS, CYP71AV1 and DBR2 genes (Ma et al., 2009; Han et al., 2014; Jiang et al., 2016). Overexpression of a trichome-specific WRKY from *Artemisia annua*, named AaGSW1, significantly improves the contents of artemisinin and dihydroartemisinic acid by regulating CYP71AV1 and AaORA expression (Chen et al., 2017b). In addition, it has been reported that GaWRKY1 of *Gossypium arboretum* regulates sesquiterpene synthase in the biosynthesis pathway of sesquiterpene phytoalexins, including gossypol (Xu et al., 2004). Although some WRKY TFs have been identified in plants, there are no studies on WRKY TFs from *A. paniculata*. Andrographolide is a diterpenoid lactone derived from the terpene skeleton pathway. Therefore, we proposed that WRKY TFs may be involved in andrographolide biosynthesis.

The genome-wide identification of WRKY TFs has been documented in some plants, such as the model plant *Arabidopsis thaliana* (72); the important crops *Oryza sativa* (102), *Zea mays* (136), *Glycine max* (182), and *Cucumis sativus* (55); and the model medicinal plant *Salvia miltiorrhiza* (61) (Wu et al., 2005; Ling et al., 2011; Wei et al., 2012; Li et al., 2015). Although *A. paniculata* is a famous and widely used medicinal plant, its WRKY TFs has not been studied. This study aims to provide a more comprehensive analysis of the WRKY family in *A. paniculata*. In total, 58 WRKYs were identified in *A. paniculata*. Furthermore, analyses of chromosome locations, sequence alignment, the phylogenetic tree, gene structure, conserved motifs, GO annotation, synteny, and alternative splicing events were performed. To infer the function of WRKY genes regulating andrographolide biosynthesis, *cis*-element prediction, differentially expressed genes (DEGs) analysis, qRT-PCR detection, and phylogenetic analysis were applied in this study. Finally, we suggested seven WRKYs that may be involved in the biosynthesis of andrographolide, which laid the foundation for further regulatory research.

## Materials and Methods

### Plant Materials and Treatment

In this study, the plants of *A. paniculata*, which were pot cultivated in the laboratory at the School of Pharmaceutical Sciences of Guangzhou University of Chinese Medicine (China), were used. The healthy and full *A. paniculata* seeds were cultured in petri dishes for 10 days under 25°C until cotyledons grew. Then the seedlings were transplanted into flowerpots. For methyl jasmonate (MeJA) treatment, 20-day seedlings in flowerpots were used and each group (*n* = 3) was sprayed with 5 mM MeJA. The samples were collected at 0, 24, and 48 h after spaying from nine individual plants. For different organ samples, roots, stems, and leaves were collected from three plants grown for 45 days. All materials were immediately frozen in liquid nitrogen and stored at −80°C for analysis of the expression patterns of WRKY genes.

### Database Search and Gene Identification

The WRKY sequences of *A. thaliana* were obtained from the *Arabidopsis* Information Resource (http://Arabidopsis.org/) and used as queries to search the *A. paniculata* genome database (Sun et al., 2019). Then, we corrected the sequences manually using the BLASTx algorithm (http://blast.ncbi.nlm.nih.gov/Blast.cgi) by comparison with other plant WRKYs. All WRKY sequences were further confirmed with PROSITE (http://prosite.expasy.org/), and the conserved domains of each WRKY protein were obtained.

### Phylogenetic, Gene Structure, Conserved Motif Analyses, and Chromosomal Locations

The WRKY domain amino acid sequences of *A. paniculata* and *A. thaliana* were aligned with ClustalX program by MEGA 7.0 software, and phylogenetic analysis was carried out using the neighbor-joining method with 1,000 bootstrap replicates. The molecular weights (MWs) and isoelectric points (pIs) of the putative WRKY proteins were calculated by the ExPASy proteomics server (https://web.expasy.org/protparam/). The gene exon-intron organization was constructed using Gene Structure Display Server (v2.0 http://gsds.cbi.pku.edu.cn/). Motifs in all ApWRKY protein sequences were identified by the MEME 5.0.1 online program (http://meme-suite.org/tools/meme) with the following parameters: number of repetitions, any; maximum number of motifs, 15; and the optimum width of each motif, between 20 and 50 residues. The figure was represented using TBtools software (Chen et al., 2020). The corresponding chromosomal locations of WRKY genes in the *A. paniculata* genome were downloaded from the *A. paniculata* genome database. The WRKY gene locations were then represented using Mapchart software.

### Synteny Analysis, GO Annotation, Alternative Splicing Analysis, and *Cis*-element Prediction

The alignment was performed by LASTZ using CDS sequences of *A. paniculata* and four representative species (*A. thaliana, Oryza sativa, Vitis vinifera*, and *Sesamum indicum*). The syntenic block map was constructed by MCscan with cscore = 0.7. The gene ontology annotation of WRKY amino acid sequences was analyzed using Blast2GO (http://www.blast2go.com). The sequences were screened by BLASTp against the InterProScan database and NR database. After mapping to the GO term, the annotation was conducted with default parameters. Alternative splicing analysis was performed as in our previous study (Gao et al., 2019). The 2,000-bp promoter sequences of 56 genes encoding key enzymes in the andrographolide biosynthetic pathway were obtained from the *A. paniculata* genome database. Place (http://www.dna.affrc.go.jp/PLACE/signalscan.html) was used to identify the *cis*-elements by WRKY TFs.

### Gene Expression Analysis and qRT-PCR

The RNA-seq reads from four organs (root, stem, leaf, flower, fruit) and MEJA-treated seedlings (0, 24, 48) were generated and the expression patterns of the WRKY genes were analyzed with TopHat and Cufflinks (Trapnell et al., 2012). According to the manufacturer's instructions, total RNA was extracted from each sample with an RNAprep Pure Plant Kit (Tiangen Biotech, Beijing, China). The quality of the RNA was detected by electrophoresis and a NanoDrop 2000C spectrophotometer. Total RNA was reverse transcribed into the first cDNA strand using a FastQuant RT Kit (Tiangen Biotech). The gene-specific primers were designed by Primer Premier 6.0, and the PCR product size was set between 130 and 200 bp (Supplementary Table 1). ApActin was used as the reference gene. qRT-PCR was performed using TaKaRa TB Green^TM^ Premix Ex Taq^TM^ II (Dalian, China) on an Applied Biosystems 7500 Fast Real-Time PCR System. Three technical replicates were conducted for each sample. Statistical analysis was carried out to detect expression differences using IBM SPSS 20 software, and a *P*-value < 0.01 was considered extremely highly significant.

## Results

### Identification of the *A. paniculata* WRKY Family and Its Chromosomal Distribution

**Figure 1:**
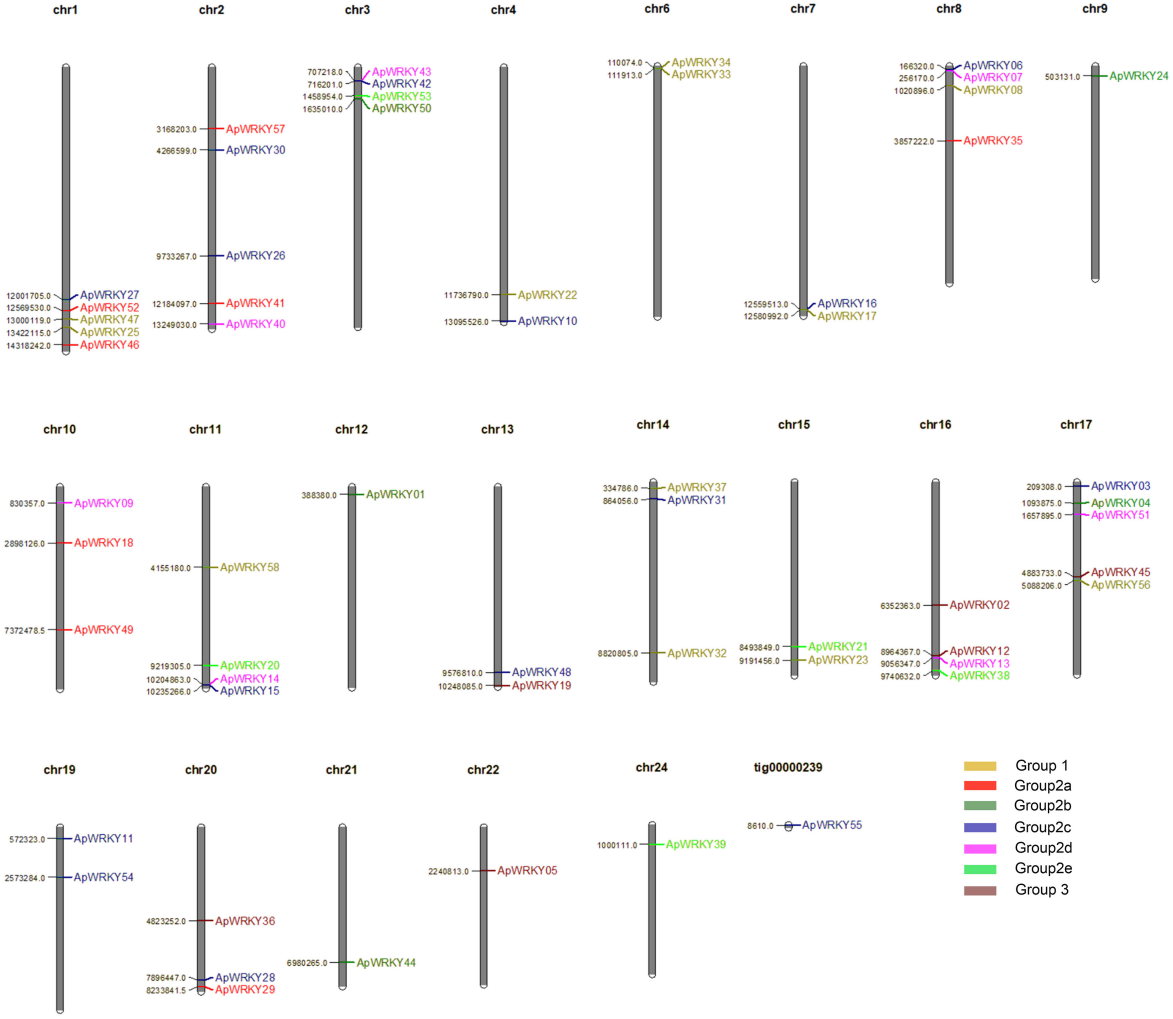
Distribution of 58 WRKY genes on the 21 Chuanxinlian chromosomes and one tig. Different group members are presented in various colors: yellow, Group 1; red, Group 2a; dark green, Group 2b; blue, Group 2c; pink, Group 2d; light green, Group 2e; brown, Group 3.

A total of 58 WRKY genes were identified from the genome database of *A. paniculata*, and the WRKY domain was further confirmed by PROSITE software. The gene chromosomal location analysis revealed that 57 WRKY genes were located on 21 chromosomes (Figure 1). The remaining 1 gene, WRKY55, was located at tig00000239. There was an average of 3 WRKY genes per chromosome, with the highest number of genes (five genes) located on chromosomes 1, 2, and 17. In addition, the lengths of the genomic DNA, cDNA, and the deduced protein; the domain sequence; the number of introns; and the physicochemical properties (molecular weight, isoelectric point, grand average of hydropathicity and aliphatic index) are summarized in Supplementary Table 2.

### Classification of the WRKY Domains and Phylogenetic Analysis

**Figure 2:**
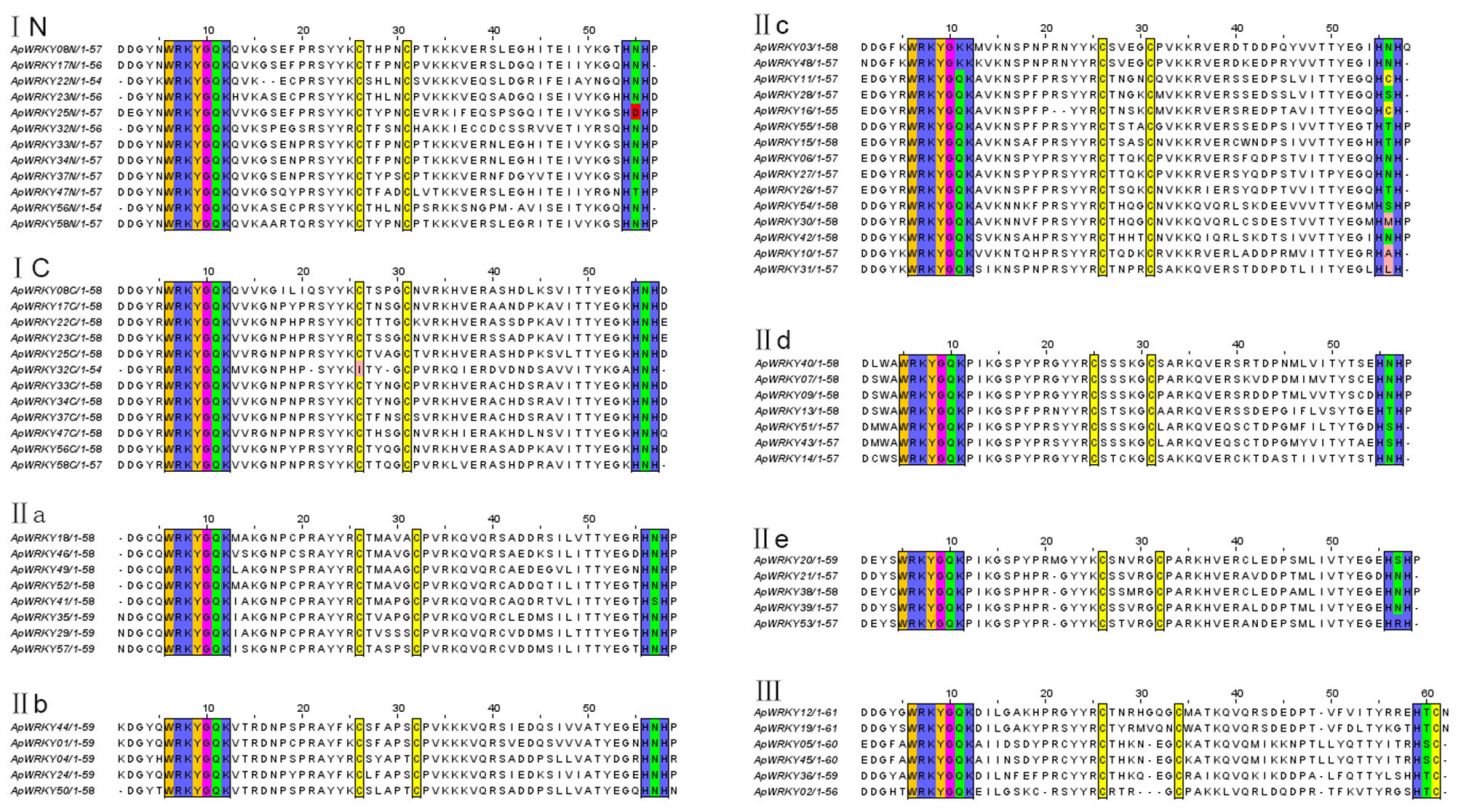
Comparison of deduced amino acid sequences of the WRKY domains from ApWRKYs. Black box indicates conserved WRKY amino acids and zinc-finger motif.

**Figure 3:**
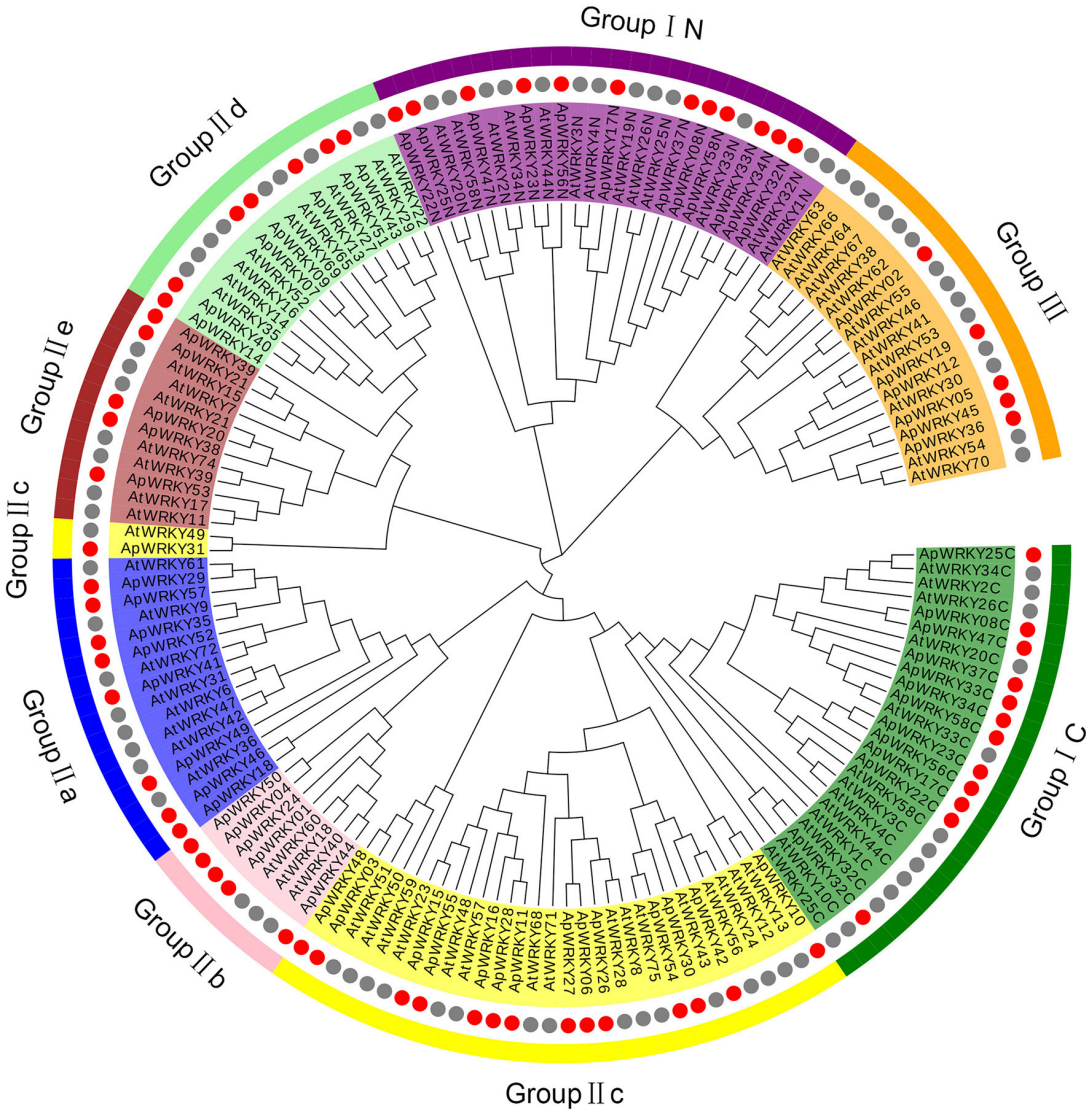
Phylogenetic relationships of WRKY proteins from *A. paniculata* and *Arabidopsis* using WRKY domain sequences. Groups are highlighted.

The WRKY domain sequences of *A. paniculata* and *A. thaliana* were aligned with the default settings, and phylogenetic relationships were analyzed using the neighbor-joining algorithm by MEGA7.0 software with 1,000 bootstrap sampling steps (Figures 2, 3). The result of the WRKY domain sequence alignment indicated that each domain possessed ~60 residues, including the highly conserved motif WRKYGQK and a zinc finger motif. Among them, 57 domains contained the highly conserved sequence WRKYGQK, while only one had WRKYGKK (Figure 2). According to the classification of WRKYs from *A. thaliana*, the ApWRKYs were divided into three groups (Figure 3). Group I contained 12 members with two WRKY conserved domains, which were further designated the N-terminal WRKY domain (NTWD) and the C-terminal WRKY domain (CTWD). Forty members were clustered in Group II and possessed only one WRKY conserved domain. Based on the primary amino acid sequence, Group II proteins can be further divided into five subgroups (IIa-IIe). In addition, six WRKYs with one WRKY domain were identified in Group III. Group I and Group II harbored a C2H2 zinc finger motif, while Group III had the other zinc finger motif (C2HC).

### Gene Structure and Conserved Motifs of WRKYs

**Figure 4:**
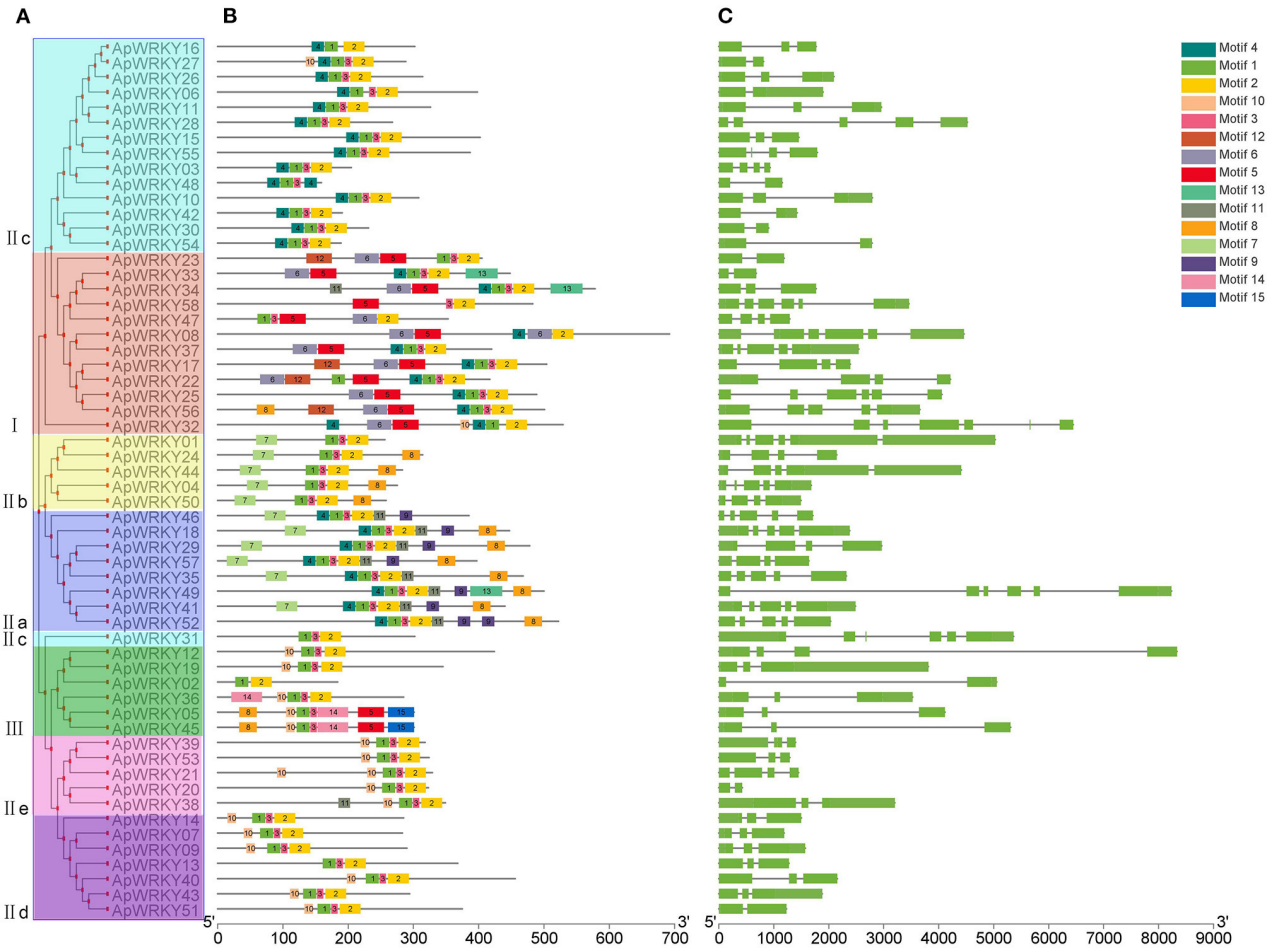
The gene structure and the distribution of conserved motifs within each ApWRKY in *A. paniculata*. **(A)** Phylogenetic tree of ApWRKY proteins. **(B)** The distribution of conserved motifs within each ApWRKY protein. **(C)** The gene structures of *ApWRKYs*. Exons were represented by green boxes and introns were represented by black lines.

Conserved motifs are sometimes associated with specific functions such as transcription repression of EAR motif and R/KLFGV motif; transcriptional activation of EDLL motif and WRKY-protein interaction of VQ motif (Ohta et al., 2001; Ikeda and Ohme-Takagi, 2009; Tiwari et al., 2012; Chi et al., 2013). In this study, we used the MEME tool to predict the conserved motifs within the WRKY protein sequences, and a total of 15 motifs were obtained (Figure 4B and Supplementary Table 3). Some motifs were highly conserved and were distributed in all the proteins, while some specific motifs appeared only in certain groups (Figures 4A,B). For example, all WRKYs had motif-1, motif-2, and motif-3. However, motif-11, motif-14 and motif-15 existed only in Group I. Motif-7 and motif-9 were found only in Group III, and only Group IIa possessed motif-13. Moreover, motif-12 specifically belonged to Group IIe and Group IId. The information of these particular conserved motifs provided clues for further study on protein structure, function and evolution.

The gene structure map showed that the number of introns in WRKY genes of *A. paniculata* varied from 1 to 5, and only two genes (*ApWRKY06* and *ApWRKY30*) from Group IIc were intronless (Figure 4C). Members of Group I generally had more introns than others; for example, *ApWRKY25, ApWRKY32, ApWRKY33*, and *ApWRKY58* contained up to five introns.

### Go Ontology Annotation and Synteny Analysis of WRKY Genes

Blast2GO (http://www.blast2go.com) was used to annotate all the WRKYs. Gene Ontology consists of three categories: molecular function, biological process and cell component. The “binding” term, belonging to molecular function, appeared the most frequently, and 24 WRKYs were annotated with it (Supplementary Figure 1). In the biological process category, only three sequences were assigned to “response to stimulus.” Moreover, the remaining WRKYs were evenly distributed across the molecular function, biological process and cellular component categories with 12–13 sequences.

**Figure 5:**
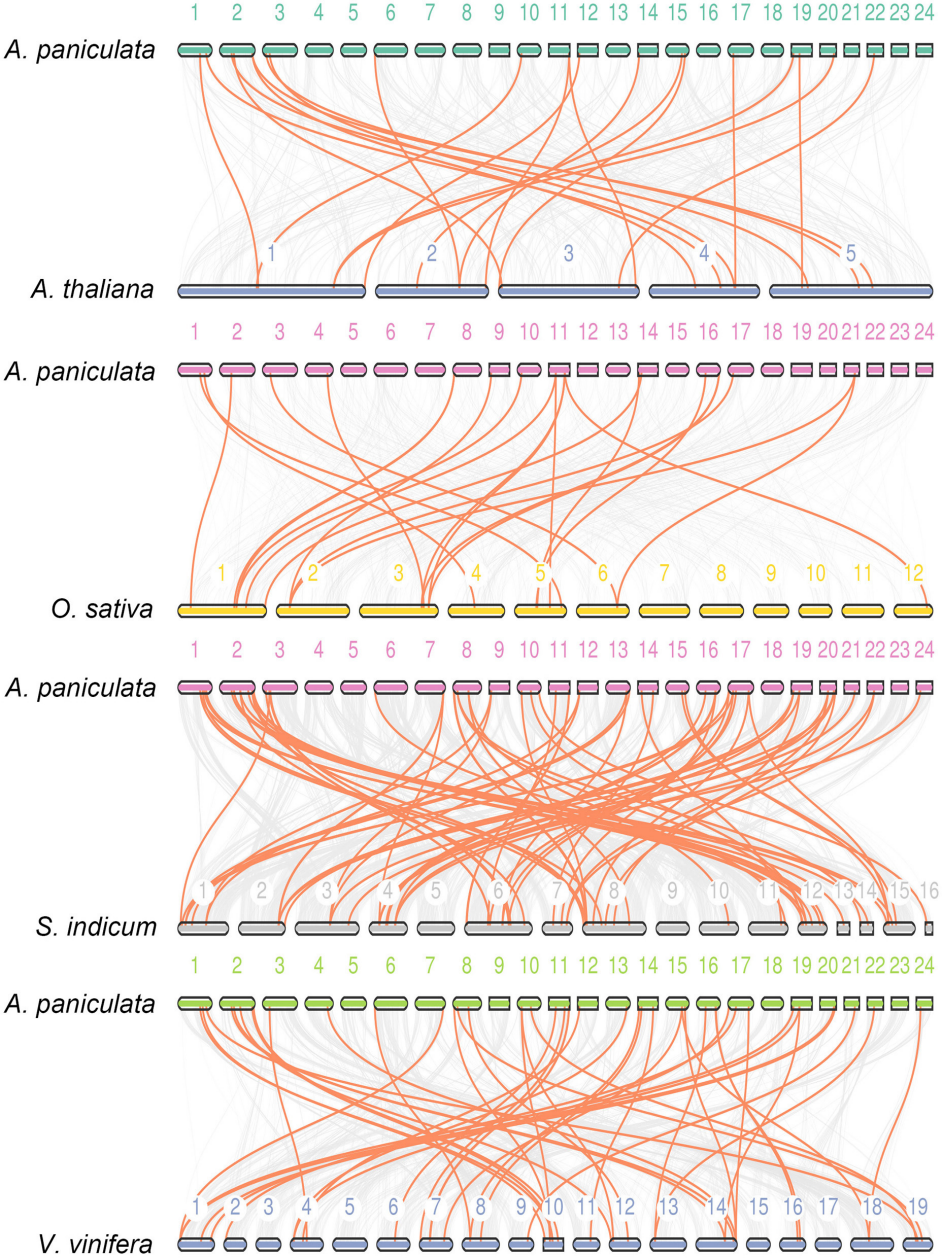
Synteny analysis of *WRKY* genes. Synteny between *A. paniculata* and *Arabidopsis, O. sativa, S. indicum* and *V. vinifera* is shown. Colored lines denote syntenic regions between Chuanxinlian chromosomes and others.

Synteny is part of the important reference information for investigating the evolutionary history of gene families and exploring functional genes (Ksiazkiewicz et al., 2017; Raboanatahiry et al., 2017; Zhao et al., 2017; Zhang et al., 2018; Hardigan et al., 2020). In this study, we used three dicotyledonous plants comprising *A. thaliana, Vitis vinifera* and *Sesamum indicum* and one monocotyledonous *O. sativa* to construct a synteny relationship map with *A. paniculata* (Figure 5). The figure indicates that the collinear relationship between *A. paniculata* and *S. indicum* and *V. vinifera* was the most significant, while the gene pairs between *A. paniculata* and *O. sativa* were the least significant. These results provide clues for examining the relationship between functional genes.

### Analysis of Alternative Splicing Events

**Figure 6:**
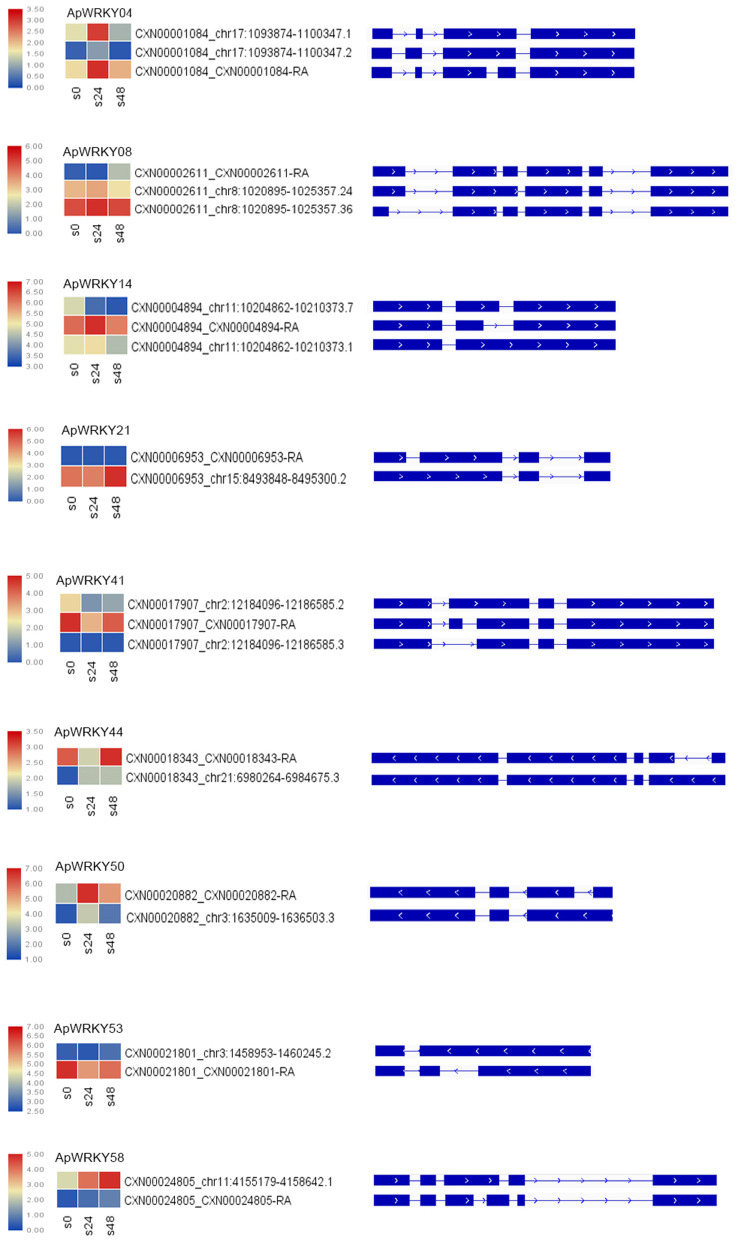
Alternative splicing isoforms of nine *ApWRKY* genes. Heatmap of the isoforms showed changes in the gene expression of various isoforms under MeJA stress.

The main types of alternative splicing events include exon skipping, intron retention, mutually exclusive exons, A5SS (alternative 5' splice site) and A3SS (alternative 3' splice site) (Chaudhary et al., 2019). Based on the isoform detection and prediction (IDP) results of this study, 22 splicing events of 9 WRKY genes were identified (Figure 6). The splicing types included 12 IR isoforms, six non-IR isoforms, and nine ref isoforms. Five genes, including *ApWRKY21, ApWRKY44, ApWRKY50, ApWRKY53*, and *ApWRKY58*, had two splicing types. The remaining four genes presented three splicing types. After treatment with MeJA for 24 h, the expression levels of the isoforms from most genes were upregulated. For example, IR isoforms and ref isoforms produced by *ApWRKY04, ApWRKY14*, and *ApWRKY50* were all induced. Moreover, after MeJA treatment for 48 h, the expression levels of various alternative splicing types varied. The expression of IR isoforms from *ApWRKY21* and *ApWRKY58* constantly increased within 48 h. However, the expression levels of the reference and IR isoforms of *ApWRKY50* and *ApWRKY04* were decreased at 48 h compared with 24 h. Alternative splicing is widespread in eukaryotic organisms, which leads to polymorphisms in the structure and function of proteins (Chaudhary et al., 2019). The complexity of these alternative splicing events may play an important regulatory role in the Chuanxinlian development-mediated MeJA pathway.

### Expression Patterns of WRKYs in *A. paniculata*

**Figure 7:**
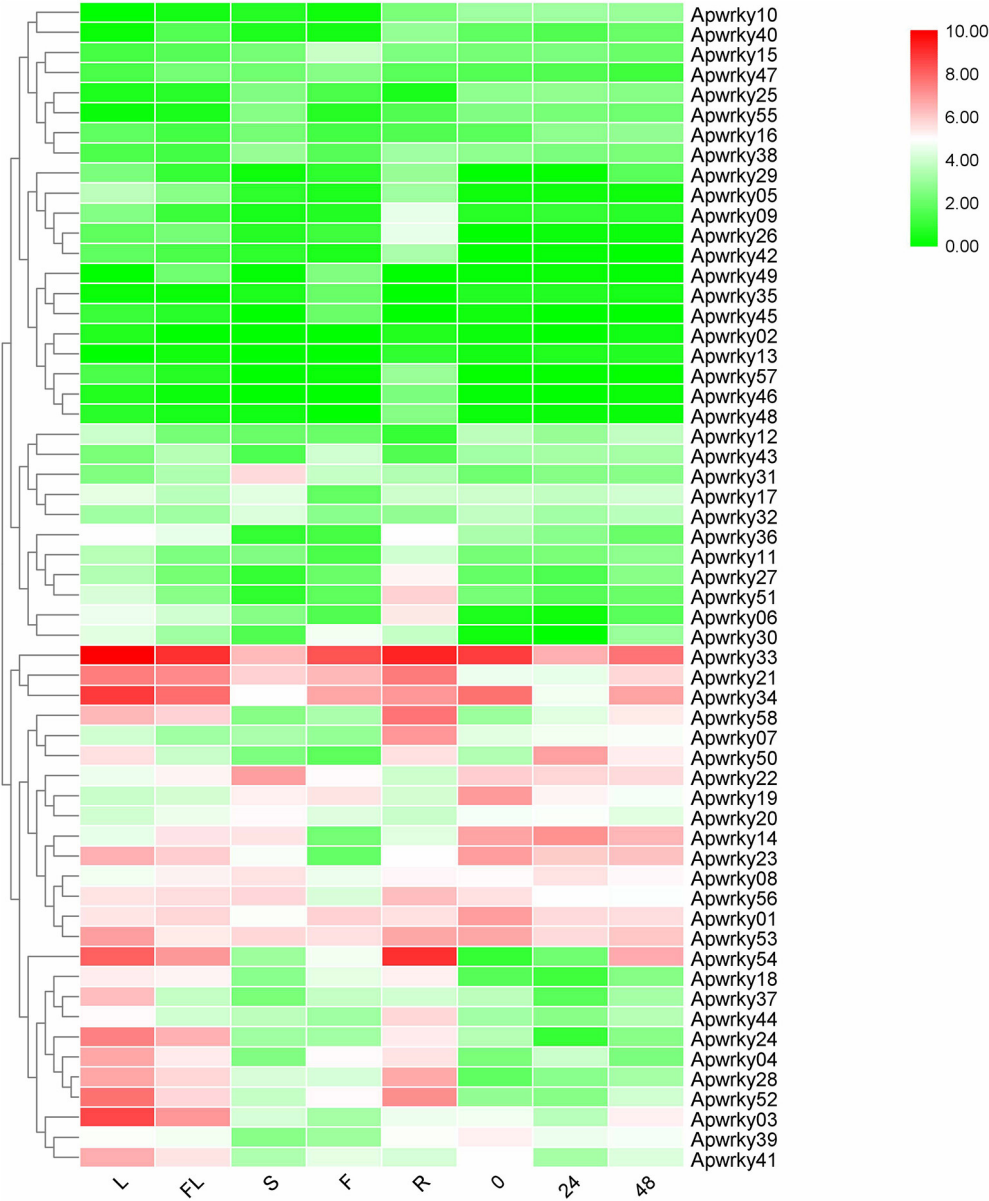
Heatmaps representing the expression profiles of Chuanxinlian *ApWRKY* genes in leaf, root, stem, flower, fruit and MeJA-treated seedings.

Different expression profiles of genes in various organs and stresses can supply essential information for screening for functional genes. The expression levels of *WRKYs* in *A. paniculata* were calculated using RNA-seq data (Figure 7 and Supplementary Table 4). *ApWRKY02* and *ApWRKY13* were not expressed in all organs (FPKM value >1), and other genes were expressed at different levels among the organs of flowers, fruits, roots, stems and leaves. A total of 16 genes were most highly expressed in leaves (FPKM value >10); and 5, 12, and 5 genes in fruits, roots, and stems were most highly expressed (FPKM value >10), respectively. These genes may participate in organ development in *A. paniculata*. The expression profiles of 58 *WRKYs* in MeJA-treated seedlings were also observed. After MeJA treatment for 24 h, the expression of 12 *WRKYs* was upregulated compared with the expression at 0 h. After 48 h of MeJA treatment, 4 *WRKYs* were induced consistently within 24 h. For example, the expression profile of *ApWRKY58* increased from 7.12 to 41.57. These results provide transcriptome reference information for us to reasonably speculate on genes that may be responsible for the development and tolerance regulation of *A. paniculata*.

### WRKY Transcription Factors Associated With Andrographolide Biosynthesis

Four approaches, including WRKY binding site prediction, differential gene expression analysis, coexpression analysis, and phylogenetic analysis, were performed to predict genes involved in andrographolide biosynthesis. WRKY transcription factors can specifically bind to the W-box element [(T) TGAC (C)] located in the target gene promoter (Cheng et al., 2019; Liu et al., 2019; Zhu et al., 2019; Lv et al., 2020). In our study, the 2.0 kb promoter sequences of 56 key enzyme genes in the andrographolide biosynthetic pathway were analyzed, and a total of six binding sites were identified, of which WRKY71OS was possessed by all key enzyme gene promoters (Supplementary Table 5). The promoters of various genes possessed different binding sites. *HMGR1* possessed six types of binding sites, and *HMGS1* possessed three types of binding sites. These results imply that WRKYs may bind directly to W-box elements to regulate the expression of key enzyme genes and thereby affect andrographolide biosynthesis in *A. paniculata*.

**Figure 8:**
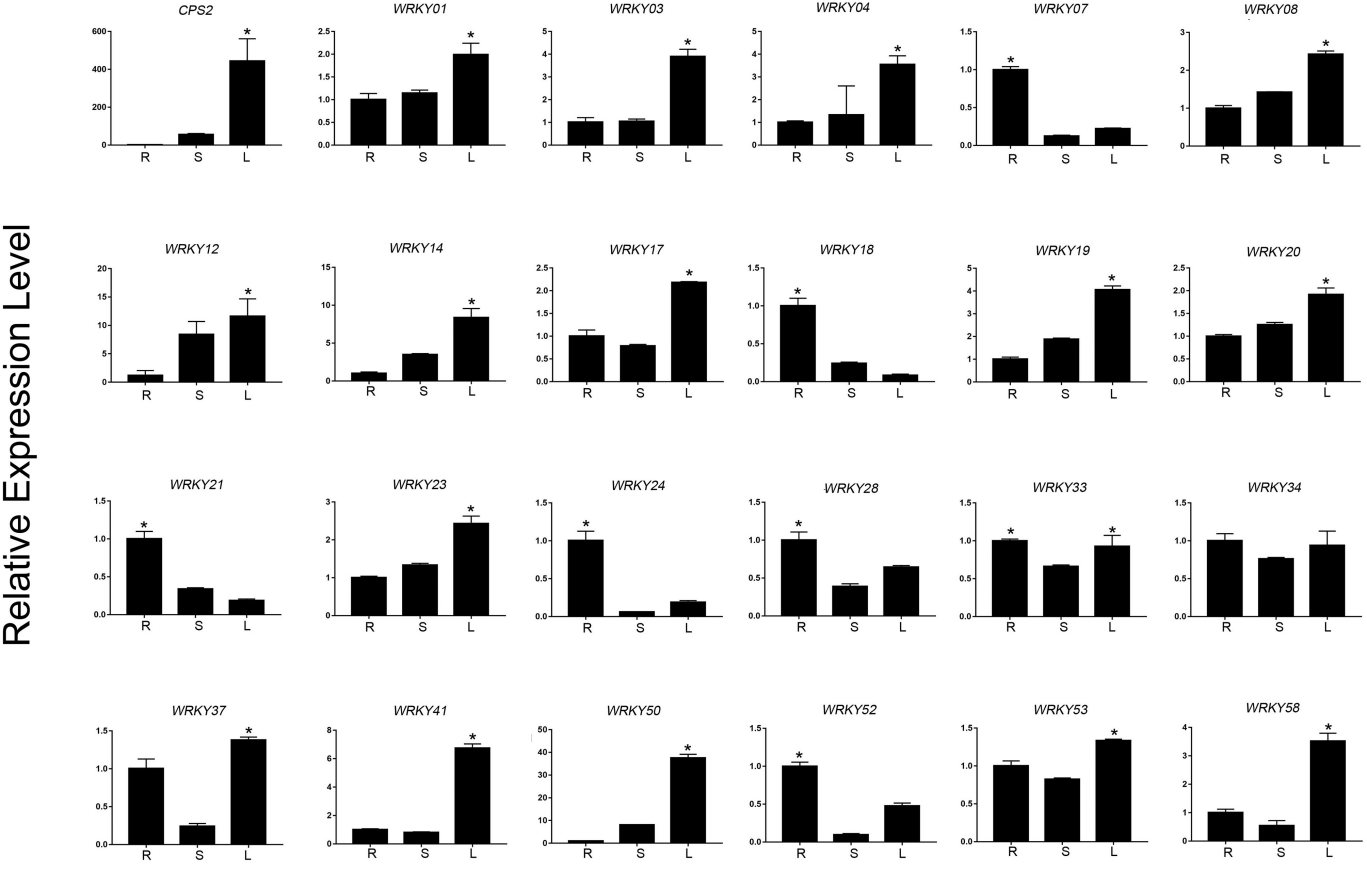
qRT-PCR analysis of *ApWRKYs* with putative roles in andrographolide biosynthesis in different organs (root, stem, leaf) in *A. paniculata*. The actin gene was tested as an internal reference. *CPS2* was used as the positive control. The asterisks (*) represent significant differences (*P* < 0.05).

**Figure 9:**
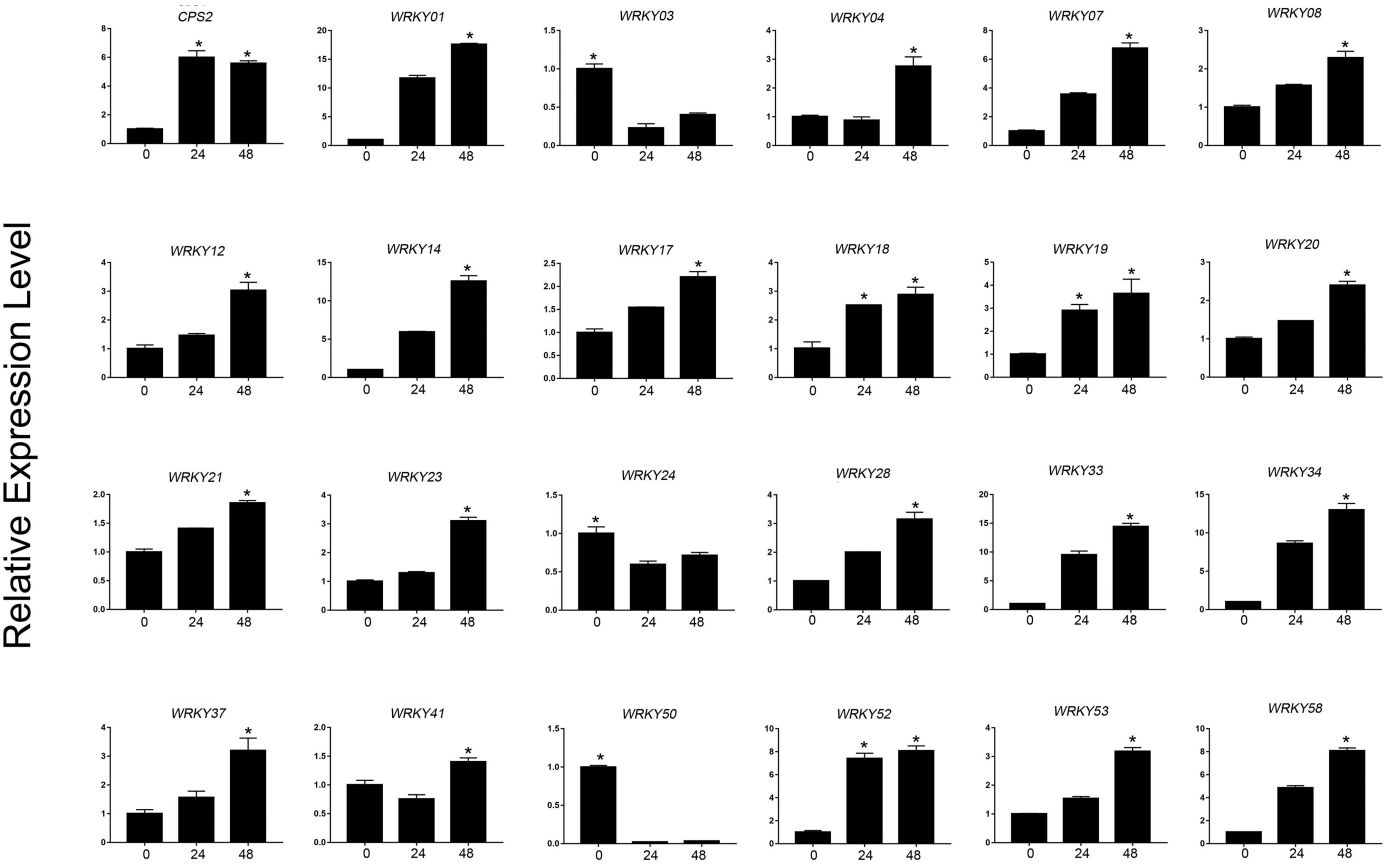
qRT-PCR analysis of *ApWRKYs* with putative roles in andrographolide biosynthesis in MeJA-treated seedlings (MeJA-24 and MeJA-48, respectively) in *A. paniculata*. The actin gene was tested as an internal reference. *CPS2* was used as the positive control. The asterisks (*) represent significant differences (*P* < 0.05).

Based on the expression level of WRKYs derived from the RNA-seq data, we preliminarily selected 24 WRKY genes, and their expression was further confirmed using qRT-PCR (Figures 8, 9). The qRT-PCR results showed that six genes (*ApWRKY01, ApWRKY08, ApWRKY12, ApWRKY14, ApWRKY19*, and *ApWRKY20*) were both most highly expressed in leaves and MeJA-treatment seedlings. Notably, another gene *ApWRKY50* was most highly expressed in leaves, followed by stems, and lowest in roots. The expression level of *ApWRKY50* was nearly 40 times higher than in roots, similar to that of the key enzyme gene *CPS2*. Though it was downregulated by MeJA, it seemed that ApWRKY50 maybe participate in the biosynthesis of andrographolide in leaves. A phylogenetic tree was further constructed using protein sequences of the candidates and the functional WRKYs known to regulate secondary metabolism. The seven WRKYs were all clustered with the functional WRKYs that regulated terpene biosynthesis. For instance, ApWRKY12 and ApWRKY19 were clustered with AaWRKY1, which regulate artemisinin from *A. annua* (Ma et al., 2009; Han et al., 2014). ApWRKY01 and ApWRKY50 belonged to Clade 2 with GaWRKY01 from *Gossypium arboreum*, AtWRKY40 and AtWRKY18 from *A. thaliana* (Xu et al., 2004; Alfieri et al., 2018). Therefore, based on the sensitive and tissue specific gene expression manner and evolutionary relationship of WRKYs, we suggested that the seven candidates are likely to participate in the biosynthesis of andrographolide.

## Discussion

Medicinal herbs have been widely used clinically due to their natural origins, rich variety, and low toxicity. *A. paniculata* is a well-known Chinese medicine in China and South Asian countries, and andrographolide, as the main bioactive component of *A. paniculata*, has many pharmacological activities. As the demand for andrographolide has grown over the past years, obtaining higher contents of andrographolide by controlling the biosynthesis pathway of andrographolide has attracted researchers' attention. Transcription factors are important regulators that can activate biosynthetic enzyme genes at the transcription level to promote active compound production (Bai et al., 2018; Cao et al., 2018; Pan et al., 2019). In *A. paniculata*, transcription factors have rarely been studied. Therefore, we report work relevant to the WRKY TFs in the regulation of andrographolide biosynthesis. This represents a first major step for the further identification of WRKYs involved in andrographolide biosynthesis.

Some special motifs may provide certain reference information for investigating genes function. The conserved motif distribution in all 58 WRKYs was obtained using MEME online software. Motif 1, motif 2 and motif 3, motif 5, motif 6 and motif 14 were all within the WRKY domain region, among which motif 1 was the core WRKY motif (WRKYGQK). Motif 2, motif 3 and motif 4 were assigned to a complete C2H2-type zinc finger structure. In the 58 ApWRKYs, 16 proteins have the EAR (ERF-associated amphiphilic repression) motif, which appears in the form of LxLxL or DLNxxP, was the first active repression motif reported in plants with diverse biological functions (Kagale and Rozwadowski, 2011; Shyu et al., 2012; Rakesh et al., 2014). Among the 16 ApWRKY proteins, only 3 ApWRKYs have DLNxxP-type EAR motifs. The remaining 13 proteins all possessed the LXLXL-type EAR motif. Moreover, 8 ApWRKY proteins (ApWRKY5, ApWRKY6, ApWRKY24, ApWRKY39, ApWRKY42, ApWRKY45, ApWRKY50, and ApWRKY57) contain LxxLL motifs, which may participate in protein-protein interactions that can activate or repress transcription. In *Arabidopsis*, AtERF11 with an EAR motif plays a key role in affecting ethylene biosynthesis by negatively regulating the expression of AtACS2/5 (Li et al., 2011). In *Salvia miltiorrhiza*, SmJAZ8 has an LXLXL-type EAR motif at the N terminus and negatively regulates the biosynthesis of salvianolic acids and tanshinones by reducing the expression of the enzyme genes of both biosynthetic pathways (Pei et al., 2018). The ApWRKYs possessed these specific motifs may have similar functions in development, stress response as well as secondary metabolism.

Considerable studies demonstrate that “multi-omics”-based method was an efficient tool to discovery candidate genes involving plant development, stress-response, and specialized metabolism. For example, metabolome and transcriptome data of 20 tomato tissues and stages were integrated; and a new transcription factor SlERF.G3-like was co-expressed with flavonoid compounds and biosynthetic genes. Further investigation revealed SlERF.G3-like positive regulated flavonoid biosynthesis in tomato (Li et al., 2020). In our previous study, the AP2/ERF transcription factor family of *Salvia miltiorrhiza* was analyzed via a genome-wide survey (Ji et al., 2016). A candidate gene SmERF128 was obtained and identified as a positive regulator promoting the production of tanshinone, which was the famous active compound treating cardiovascular diseases (Zhang et al., 2019). Andrographolide, the main medicinal component of *A. paniculata*, exhibits site specificity, which is reflected in its accumulation being most abundant in leaves and least abundant in roots (Garg et al., 2015). In addition, the andrographolide content in seedlings was significantly increased after MeJA treatment (Sharma et al., 2015; Sun et al., 2019). Previous studies have shown that many enzyme genes and transcription factors are involved in the formation of multiple secondary metabolites, and their expression patterns are significantly correlated with the distribution of active components (Lu et al., 2013; Ji et al., 2016, 2017; Huang et al., 2019; Li et al., 2019; Zhang et al., 2019). In this study, 7 WRKYs were proposed as candidates in andrographolide biosynthesis. The expression of these genes was all most highly in leaves, followed by stems and lowest in roots; and six of them were induced by MeJA. Though the expression of *ApWRKY50* did not increase after MeJA treatment, its expression in leaves was much higher than in roots. Therefore, they were likely to be involved in andrographolide biosynthesis. In addition, the phylogenetic analysis indicated that the 7 WRKYs were all grouped with the functionally characterized WRKYs, which were involved in terpenoid biosynthesis. ApWRKY19 and ApWRKY12 were in Clade 1 with AaWRKY1 from *A. annua* and AtWRKY46 from *A. thaliana*. In *A. annua*, AaWRKY1 transcription factor binds W-box of *ADS* promoter to promote the production of artemisinin; and in *A. thaliana*, AtWRKY46 is the positive regulator in the BR (brassinosteroids) pathway (Ma et al., 2009; Han et al., 2014; Jiang et al., 2016; Chen et al., 2017a). In addition, ApWRKY1 was closely related to GaWRKY1 from cotton, which participates in regulation of sesquiterpene biosynthesis (Xu et al., 2004). Moreover, ApWRKY14 and ApWRKY20 were clustered with PqWRKY1 from *Panax quinquefolius* and OsWRKY13 from rice. PqWRKY1 is a positive regulator related to triterpene ginsenoside biosynthesis, while overexpression of OsWRKY13 downregulate Momilactone A biosynthesis (Qiu et al., 2008; Sun et al., 2013). These results indicated our candidates may be involved in the biosynthesis of andrographolide in *A. paniculata*. Further functional verification experiments based on these genes are yet to be completed.

## Conclusion

In this study, a total of 58 WRKY genes were identified in *A. paniculata* genome. ApWRKYs can be divided into group I, group II (subgroup a-e) and group III supported by phylogeny, additional protein motifs, and intron/exon structures. Based on the gene expression pattern, qRT-PCR detection, and phylogenetic analyses, we obtained seven candidate genes that may participate in andrographolide biosynthesis. Therefore, based on the mentioned results, seven candidate WRKYs were inferred to be genes regulating andrographolide biosynthesis. The function of these genes is worth further exploration and may increase the production of andrographolide in metabolic engineering.

## Data Availability Statement

The raw data supporting the conclusions of this article will be made available by the authors, without undue reservation, to any qualified researcher.

## Author Contributions

AJ and LD conceived the research. RZ, ZC, LZ, and WY performed the experiment, data analysis, and wrote the paper. ZX, BL, YM, and HG contributed to transcriptome data analysis and discussion. AJ and CJ revised the paper. All authors contributed to the article and approved the submitted version.

## Conflict of Interest

YM and CJ were employed by the company Jiangxi Qingfeng Pharmaceutical Co. Ltd., Ganzhou, China. The remaining authors declare that the research was conducted in the absence of any commercial or financial relationships that could be construed as a potential conflict of interest.

## Supplementary Material

Supplementary Figure 1 GO classification of the WRKYs in *A. paniculata*.

Supplementary Figure 2 Phylogenetic analysis of candidate WRKYs and functional WRKYs from other species.

Supplementary Table 1 Primer sequences of the candidate *WRKYs* used for qRT-PCR analyses.

Supplementary Table 2 Complete list of WRKY transcription factors identified in the *A. paniculata* genome.

Supplementary Table 3 Conserved motifs identified in WRKY transcription factors from A. paniculata by MEME analysis.

Supplementary Table 4 The average FPKM values of 58 WRKY genes in different organs and MeJA-treated seedlings.

Supplementary Table 5 Regulatory elements targeted by WRKY TFs in the promoters of the enzyme-coding genes of the andrographolide biosynthesis pathway.
