# Supplementary figures and images for: Genomic Characterization of WRKY Transcription Factors Related to Andrographolide Biosynthesis in Andrographis paniculata
Source: Front Genet. 2021 Jan 18;11:601689. doi: 10.3389/fgene.2020.601689 (PMC7848199; doi:10.3389/fgene.2020.601689)

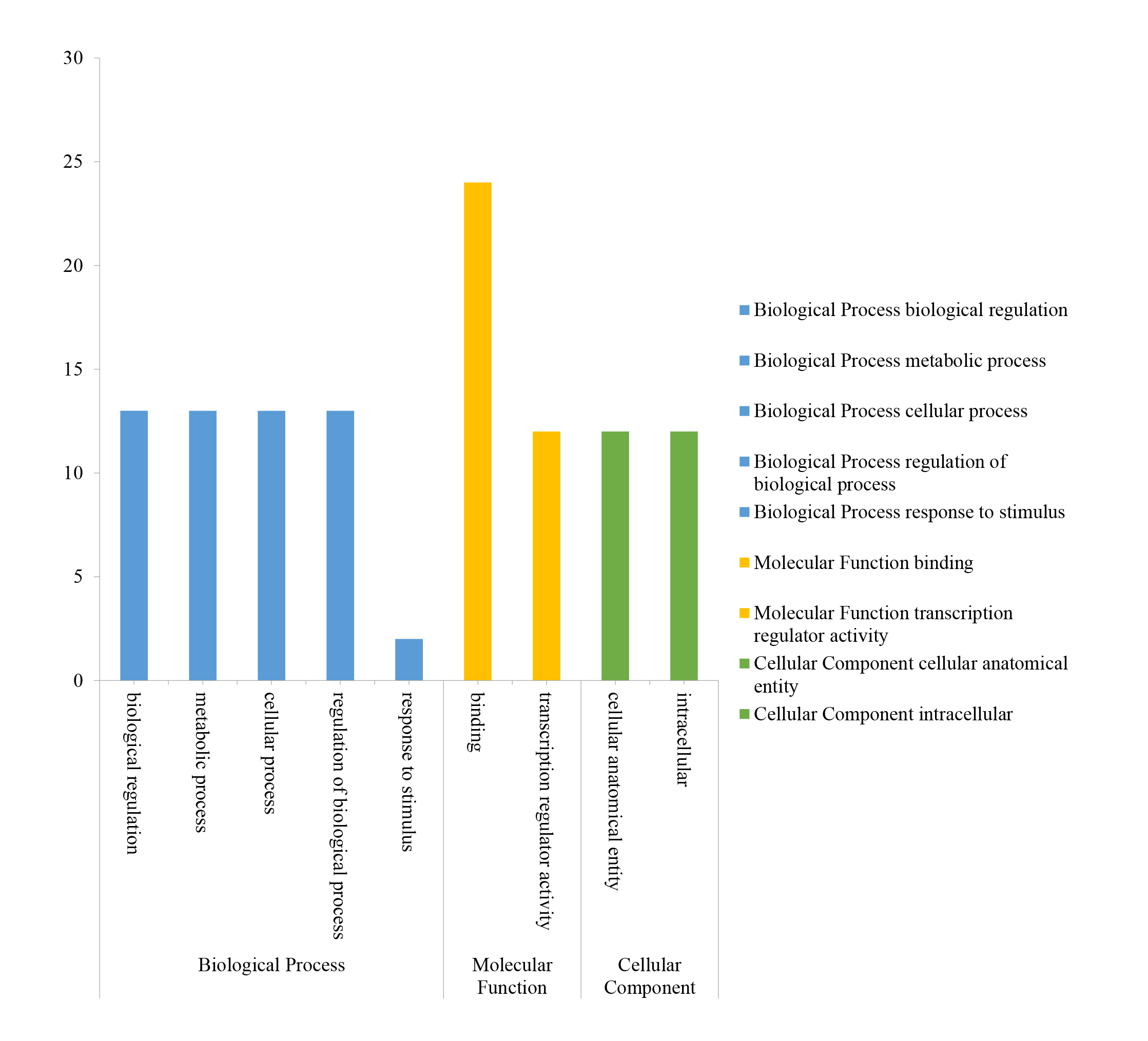

Supplement: Supplementary Figure 1 — GO classification of the WRKYs in A. paniculata. [file Image_1.JPEG]

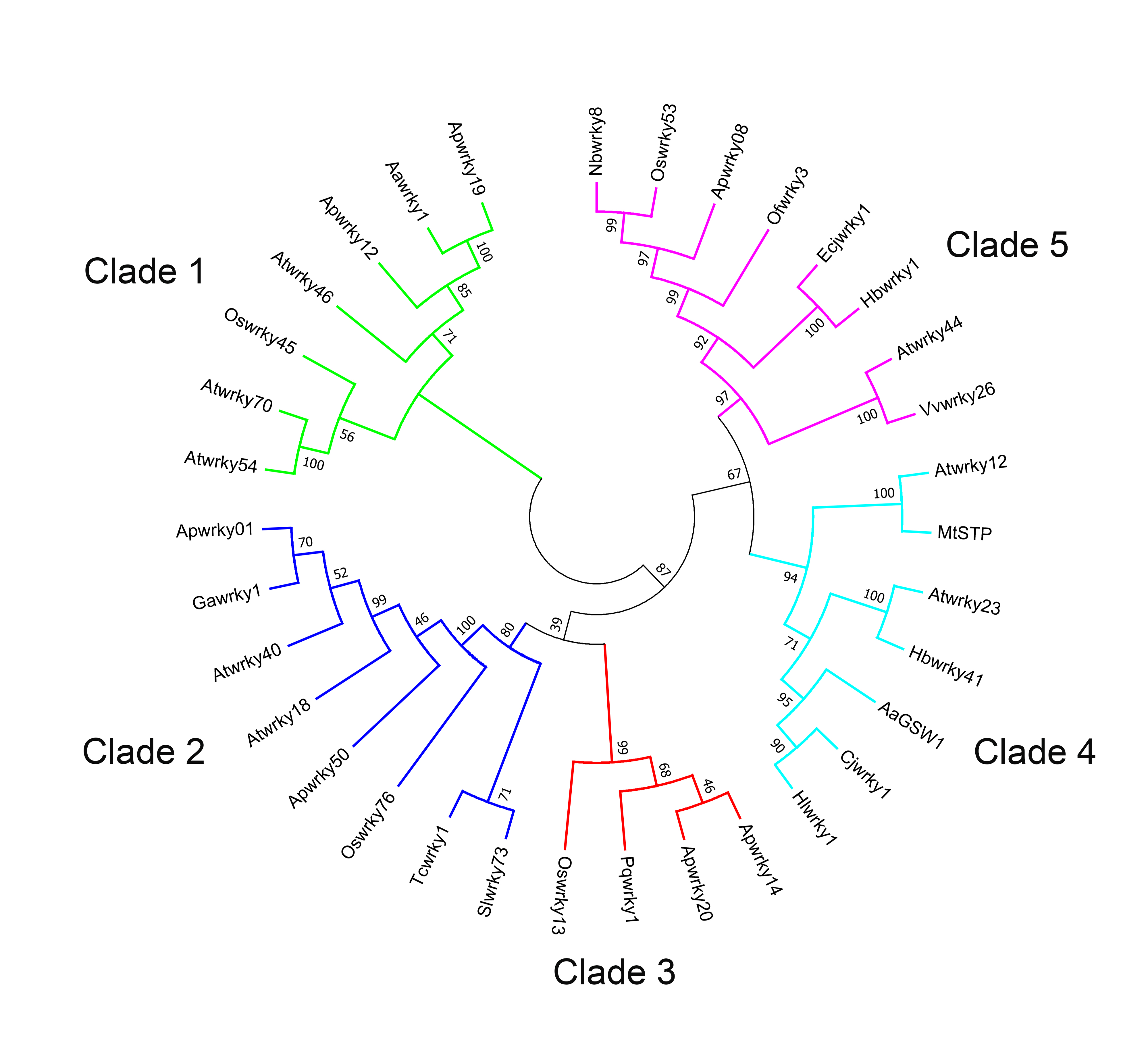

Supplement: Supplementary Figure 2 — Phylogenetic analysis of candidate WRKYs and functional WRKYs from other species. [file Image_2.JPEG]
